# Type II endometrial cancer: Incidence, overall and disease-free survival in Martinique

**DOI:** 10.1371/journal.pone.0278757

**Published:** 2023-03-16

**Authors:** Coralie Ebring, Régine Marlin, Jonathan Macni, Alexis Vallard, Sébastien Bergerac, Murielle Beaubrun-Renard, Clarisse Joachim, Mehdi Jean-Laurent

**Affiliations:** 1 Service de Gynécologie, Maison de la Femme de la Mère et de l’Enfant, CHU de Martinique, Fort-de-France, Martinique; 2 Unité de Génétique Moléculaire des Cancers du CHU Martinique, Fort-de-France, Martinique; 3 UF 1441 Registre Général des Cancers de la Martinique, Pôle de Cancérologie Hématologie Urologie, CHU de Martinique, Fort-de-France, Martinique; 4 Pôle de Cancérologie Hématologie Urologie, CHU de Martinique, Fort-de-France, Martinique; 5 Service de Médecine Nucléaire, CHU de Martinique, Fort-de-France, Martinique; Peter MacCallum Cancer Institute, AUSTRALIA

## Abstract

**Background and study aims:**

In Martinique, about 33 new cases of endometrial cancer are diagnosed per year with a high mortality rate (world standardised rate of 4.9/100,000 versus 2.3/100,000 in mainland France). The present study aimed to determine the incidence and mortality of type I and type II endometrial cancers (ECs), their overall survival (OS) and disease-free survival (DFS) between 2012 and 2016.

**Patients and methods:**

This retrospective observational cohort study used data from the Martinique Cancer Registry (MCR). 191 patients with corpus uterine cancer were extracted between 2012 and 2016. Patients with either endometrioid endometrial carcinoma (EEC), uterine papillary serous carcinomas (UPSC), uterine clear cell carcinomas (UCCC) or uterine carcinosarcomas (UCS) were included. All other uterine cancers were excluded.

**Results:**

Among the 163 included patients, 97 (60%) were type I and 66 (40%) were type II. The standardized incidence rate is 4.50/100,000 for type I vs. 2.66/100,000 for type II. Three years DFS for all types, type I and type II was 81.5% [74.2–86.9], 84.9% [75.4–91] and 76.7% [63.8–85.5] respectively. The five-years OS for all types, type I and type II was 47.0% [38.9–54.7] vs. 58.8% [47.3–68.5] vs. 22.8% [15.0–37.7] respectively.

**Conclusions:**

In Martinique, we report a high proportion of type II ECs, which has a poor prognosis with few treatment options.

## Introduction

Endometrial cancer is the fourth most common cancer in women, the most common gynecological cancer and represents 13% of all commonly occurring cancers. In France, 8,220 new cases have been identified in 2018 and the annual age-adjusted incidence per 100,000 women is 11.2 per 100,000. Its incidence increases after menopause, after which more than three-quarters of cases are diagnosed, with an average age at diagnosis of 68 years [1]. Since 2010, the incidence of endometrial carcinomas has been increasing as a result of an increase in the main risk factors, which are represented by endogenous hyperestrogenia (obesity or nulliparity), as well as therapeutic and genetic factors (Lynch syndrome) [2, 3].

Endometrial cancer is a complex and heterogeneous disease from epidemiologic, clinical, pathological and molecular standpoints. Since 1983, two pathogenic types of endometrial carcinomas were proposed [4]: (i) Type I, including the most frequent endometrial carcinomas (70% to 80%). These endometrioid carcinomas (EEC) occur most frequently at an early stage, with a low grade and a good prognosis, and a five-years survival rate of more than 85% [1, 5–7]. They consist of oestrogen driven tumors, strongly associated with obesity and other elements included in the metabolic syndrome [8, 9]. (ii) Type II, consisting of higher grade non-endometrioid tumors (20%) including uterine papillary serous carcinomas (10%), uterine clear cell carcinomas (1 to 5%) and uterine carcinosarcomas (2 to 5%). These are often diagnosed at advanced stages, with a high risk of metastatic recurrence and mortality, even when diagnosed at early stages, with few therapeutic options, and a five-years survival rate between 36% and 80% [5, 10–12]. However, this dualistic classification has become too simplistic following the development of studies on the genomic landscape of tumors. Now, different molecular profiles have been determined for these categories. Major genetic alterations associated with type I carcinomas include *PTEN*, *PIK3CA*, *PIK3R1*, *KRAS*, *ARID1A* and *CTNNB1*. An alteration in the *PIK3* signaling pathway appears to be responsible for the oncogenesis of type I endometrial tumors. Indeed, more than 50% of tumors are affected by variations in the *PTEN*, *PIK3CA* et *PIK3R1* genes [13]. Conversely, type II carcinomas often show *TP53* gene mutations, as well as *PPP2R1A*, *PIK3CA* and *FBXW7* mutations [14].

In Martinique, between 2012 and 2016, 163 new cases of endometrial cancer have been identified [15]. The Caribbean population seems to be a group at higher risk for type I endometrial cancer, due to the high prevalence of risk factors for endometrioid adenocarcinoma such as diabetes, hypertension and obesity. However, it would appear that there is also a higher incidence of type II endometrial carcinoma compared to mainland France. To date, there is no data from the Caribbean region that support this hypothesis. The only studies on populations similar to those of the Caribbean are those studying American women by ethnicity. Ethnic differences in endometrial cancer survival rates may be the result of the higher incidence of uterine papillary serous carcinoma (UPSC) seen amongst African American women. These patients had poorer survival rates when compared to white women, despite comparable surgical and systemic therapy, and after controlling for tumor histology, suggesting that other factors also contribute to the survival disparity [16–18].

The MCR is a population-based cancer registry that collects population data in Martinique, an overseas region of France with a population of 386,486 inhabitants. The MCR is one of the only two French population-based cancer registries (PBCR) among the 30 nations and territories of the Caribbean [19]. Given the incidence (world standardized rate of 7.8 per 100,000 versus 11.0/100,000 in mainland France) and mortality (world standardized rate of 4.9/100,000 versus 2.2/100,000 in metropolitan France) of endometrial cancer in people living in Martinique [1, 15] and their genotypic specificities, it is important to map the characteristics of this population. Access to this information would be a fundamental step in order to reach the goal of optimal management of patients in Martinique.

The aim of this study was to determine the incidence of type I and type II endometrial cancer in Martinique between 2012 and 2016, their overall survival and recurrence-free survival, using data from the MCR (Martinique Cancer Registry). The second aim was to determine clinical characteristics of this population, along with histo-prognostic characteristics.

## Materials and methods

### Population, design and data collection

This retrospective observational cohort study was based on data from the Martinique Cancer Registry (MCR), that included 190 patients between 2012 and 2016. Population-based cancer registries (PBCR) provide a continuous and exhaustive record of all new cancer cases occurring in the population residing in a given area, regardless of the location of diagnosis or treatment. Data were recorded in the PBCR database of Martinique in strict conformity with the international standards laid down by the International Agency for Research on Cancer, the French FRANCIM network, and the European Network of Cancer Registries. Quality control of all recorded cases in the MCR was performed in accordance with international guidelines for cancer registries. The MCR data concern patients residing in Martinique for more than 6 months. The following variables were recorded and retrieved for all cases: city of birth, date of diagnosis, patient’s age, histologic type of uterine carcinoma. Each histological report was consulted in the patients’ medical records. Patients with either endometrioid endometrial carcinoma (EEC), uterine papillary serous carcinomas (UPSC), uterine clear cell carcinomas (UCCC) or uterine carcinosarcomas (UCS) were included. All other cancers of the uterus (sarcoma, leiomyosarcoma, cervical cancer, …) were excluded.

Data were obtained retrospectively from medical records. They included demographic, clinicopathological, treatment, and outcome information. Patient characteristics consisted of age, performance status, parity, menopausal status, diabetes (yes/no), body mass index (BMI), and pathologic FIGO stage (postsurgical classification using the 2009 FIGO staging guidelines). Clinicopathological information included histological diagnosis, grade and FIGO stage (2009). The date of disease recurrence, death, or last contact was recorded.

### Statistical methods

Disease-free survival (DFS) was defined as the time from the date of diagnosis to the date of either disease recurrence or death from any cause or to the last follow-up date, which-ever occurred first. Overall survival (OS) was defined as the time from the date of diagnosis to the date of either death or the last follow-up. We collected the date of death from the files but also from match.id.fr, governmental site in which are listed all deaths. DFS and OS curves were estimated using the Kaplan–Meier method and were compared using the log-rank test. Analyses were used to assess differences between type I vs type II. P-values lower than 0.05 were considered statistically significant, indicating statistically significant differences between the two histologic cell types.

We created categories for stage of disease, early stage (FIGO IA to FIGO IB) and advanced stage (FIGO II to FIGO IV), BMI (<30; over 30 kg/m2), age (<65, ≥65 to < 75, ≥75), parity (0, 1, 2, 3, ≥4), history of diabetes (no, yes). The associations between risk factors and tumor subtypes were estimated by odds ratios (ORs) and 95% CIs using conditional logistic regression stratified jointly by categories and adjusted for age, diabetes, BMI, and parity. All *P* values were two-sided. The Cox model was used for univariate and multivariate analyses. Patients were censored at the last follow-up date, or at the cut-off date of November 7, 2015 if patients were alive at that date. For univariate survival analysis, we used the Kaplan-Meier product-limit method to estimate the proportion of survivors over time. This method allows patients with short follow-up to contribute to the survival estimate until they are censored or die, whereas higher survival estimates are contributed by patients with longer survival, albeit with a corresponding loss of precision of the survival estimates. Confidence intervals for survival estimates were computed using the Greenwood formula. The log-rank test was used to assess the statistical differences of the observed survival curves by each categorical variable: age groups, histological type, stage at diagnosis, surgery, chemotherapy, radiotherapy, brachytherapy and grade. A multivariable Cox model for censored data was performed to identify independent prognostic factors for OS [13]. Variables with a p-value <0.05 in the univariate analysis were included in the multivariable analysis. A p value<0.05 was considered statistically significant.

Statistical analyses were performed using SAS 9.4.

### Ethical aspects

The Martinique cancer registry database was approved by the French National authority for the protection of privacy and personal data (Commission Nationale Informatique et Libertés, CNIL N° 987 001). Additional approval from ethical committees was not required since our study did not involve direct patient contact.

## Results

### Patient characteristics

**Fig 1:**
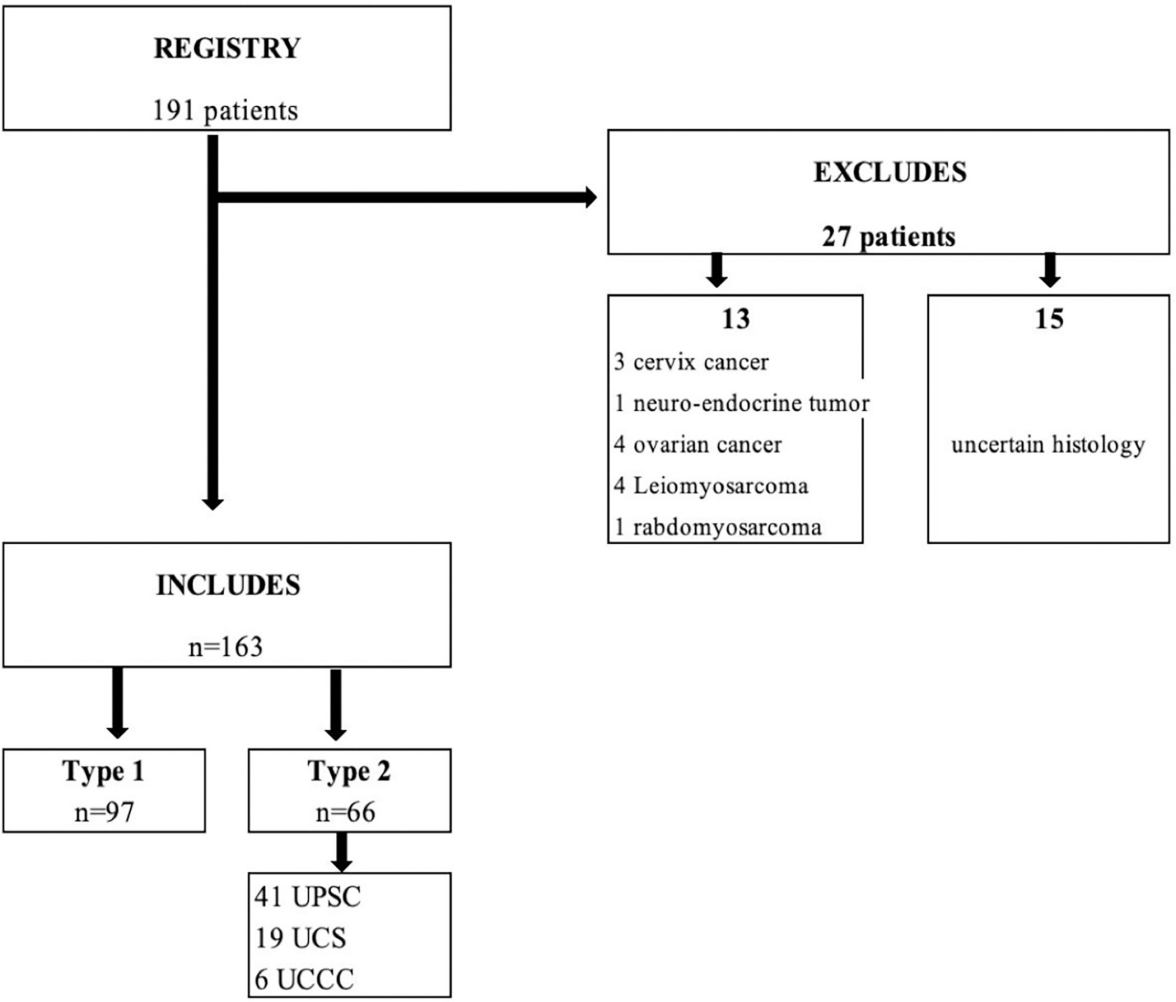
Flow chart.

In total, of the 190 patients extracted from the registry between 2012 and 2016, 163 patients were included. 28 patients were excluded, because 13 had non-endometrial cancer and 15 had a non-specific histology to distinguish between endometrioid carcinoma type I or type II (Fig 1). The patient’s clinical characteristics and comorbid conditions by cancer type are shown in Table 1. The median age at diagnosis was 69 years (range 41–95 years). The median age at diagnosis for type I and type II cancers was 67 years (range 41–95 years) and 72 years (range 57–91 years) respectively (p < 0.003). The distribution of type I and type II cancers by age group shown in Table 2. Type II represent 50% of EC diagnosed at age 75 and older, while type I represent 75% of those under age 65. UPSC and USC represent respectively 31% and 13% of those over 75 years of age. The population is comparable on diabetes and parity. The median BMI for types I and II is 30.3 kg/m2 (range 17–57 kg/m2) and 26 kg/m2 (range 16–45 kg/m2) respectively (p = 0.044). We found 49% of endometrial cancers were advanced stage with 74% of type II (p<0.001), 69% of types I were early stage (FIGO IA-IB) compared to 26% for type II (p<0.0001).

**Table 1:** Clinical characteristics and comorbid condition among patients with type 1 and type 2 endometrial carcinoma (n = 163), from the Martinique Cancer Registry.

Characteristics	All	Type 1	Type 2	*p*
n (%)	163	97 (60)	66 (40)	
Patients characteristics				
Age (Years)	69 [41–95]	67 [41–95]	72 [57–91]	<0.003
BMI (Kg/m2)	28,6 [16,5–57]	30,3 [17–57]	26 [16 – 45]	0.044
Parity	3 [0–15]	3 [0–15]	3 [0–8]	NS
Diabetes	49 (33)	27 (55)	22 (45)	NS
HTA	90 (55)	39 (43)	51 (56)	NS
Clinical stage at diagnosis	*** ***			
FIGO IA-IB	78 (51)	62 (79)	16 (21)	<0.001
FIGO II to IV	74 (49)	28 (38)	46 (62)	<0.001
Treatment	143 (87)	85 (59)	58 (41)	NS
Surgery	123 (53)	80 (65)	43 (35)	0.011
Chemotherapy	57 (35)	19 (33)	38 (66)	<0.001
Radiotherapy	75 (46)	31 (41)	44 (59)	<0.001
Brachytherapy	66 (40)	38 (58)	28 (42)	NS

Median [minimum-maximum]; n (%).

**Table 2:** Distribution of endometrial cancers (ECs) according to age groups (years).

	All	EEC	UPSC	UCS	UCCC
	*N = 163*	*n = 97*	n = 41	n = 19	n = 6
< 50	**6**	6 (100%)	0	0	0
≥ 65 to < 75	**56**	30 (54%)	15 (27%)	8 (14%)	3 (5%)
≥75	**54**	27 (50%)	17 (31%)	7 (13%)	3 (6%)

### Incidence

In total, 163 endometrial cancers were diagnosed between 2012 and 2016, an average of 33 new cases per year. The standardized incidence rate is 4.50/100,000 for Type I vs. 2.66/100,000. Type I represents 60% of the study population versus 40% for type II.

### Overall survival and disease-free survival

**Fig 2:**
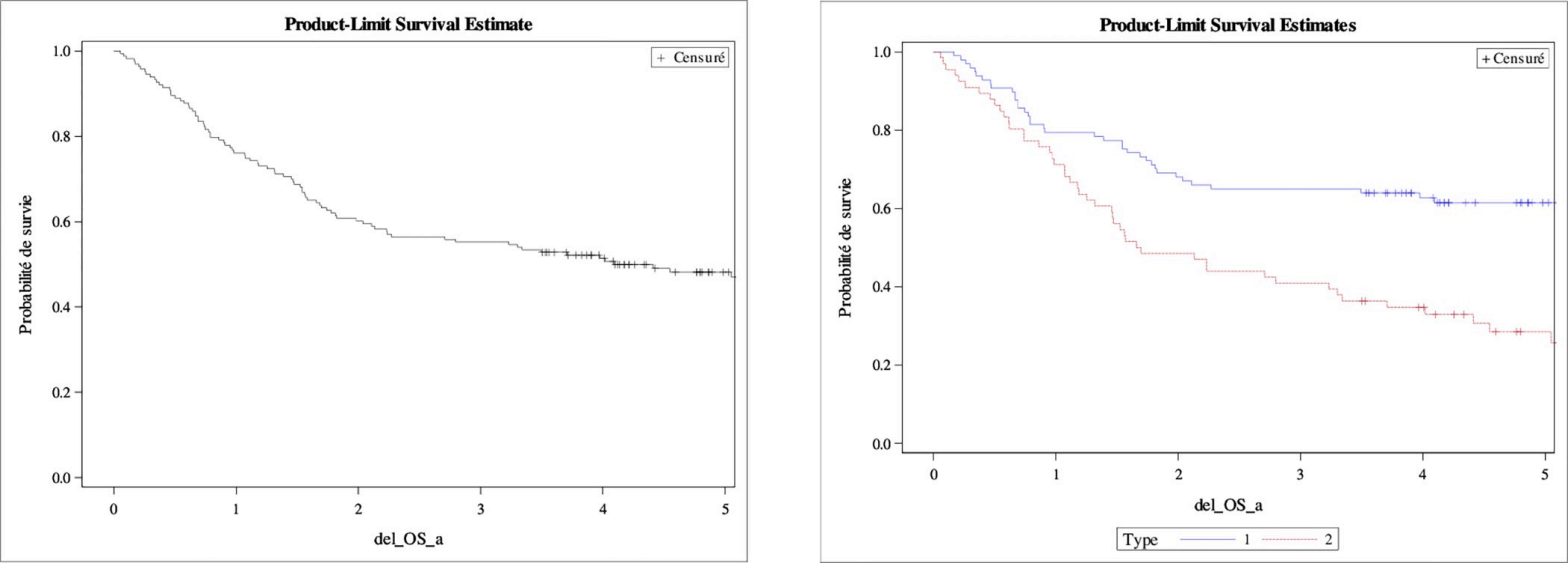
Overall survival (OS) all type, type I and type II. Kaplan-Meier estimates.

DFS and OS are represented by the Kaplan Meyer curves (Fig 2).

One-year DFS and three-years DFS for all types was 92.4% [87.4–96.0] and 81.5% [74.0–87.3]. One-year DFS and three-years DFS for type I was 90.7% [83.4–95.7] and 83.7% [75.1–90.6] respectively, vs. 93.3% [85.1–97.9] and 75.0% [63.5–85.2] for type II. We found in our multivariate model according to type of cancer that type II is twice as likely to recur.

**Fig 3:**
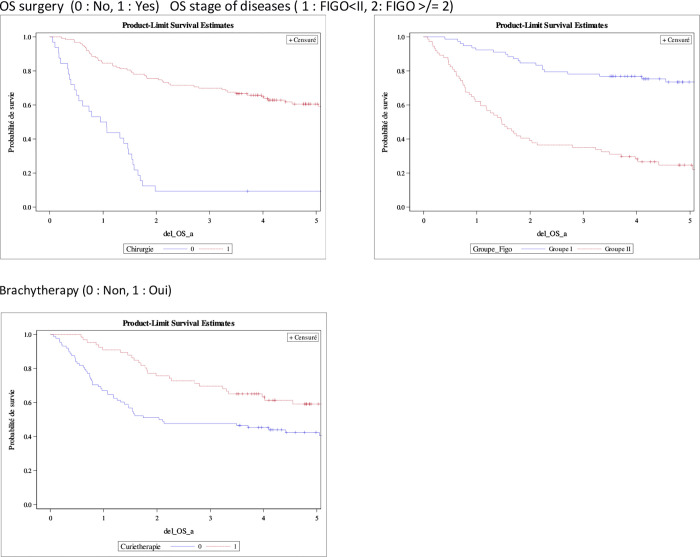
Multivariate prognostic: Risk factors of endometrial cancer survival in Martinique. Multivariate analysis.

One-year OS for all type vs. type I vs. type II was 76.1% [68.8–81.9] vs. 79.4% [69.9–86.2] vs. 71.2% [58.7–80.6], three-years OS was 55, 2 [47.3–62.5] vs. 64.9 [54.6–73.5] vs. 40.9 [29–52.4], five-years OS was 47.0% [38.9–54.7] vs. 58.8% [47.3–68.5] vs. 22.8% [15.0–37.7]. We found in our univariate analysis model that the risk factors for death are age at diagnosis (≥75 years), FIGO ≥II, type II, histo-pronostic grade ≥3, no surgery, chemotherapy, brachytherapy and radiotherapy. We found in our multivariate model, three factors that significantly influence prognosis: surgery, stage of disease at diagnosis and brachytherapy (Fig 3 and Table 3).

**Table 3:** Prognostic factors of endometrial cancer survival in Martinique, 2012–2016 (N = 163).

	Univariate HR [CI95%]	*P*	Multivariate HR [CI95%]	*p*
Global				
Histological type				
Type 1	0.41 [0.27–0.63]	<0.0001	-	
Type 2	1			
Stage of diseases				
Figo early stage (Figo = I)	0.22 [0.13–0.36]	<0.0001	0.24 [0.14–0.43]	<0.0001
Figo advanced stage (Figo>I)	1		1	
Surgery				
Yes	1	<0.0001	1	
No	5.45 [3.38–8.81]		3.21 [1.78–5.78]	0.0001
Chemotherapy				
Yes	1	<0.0001	-	
No	0.39 [0.25–0.61]			
Brachytherapy				
Yes	1	0.0060	1	
No	1.92 [1.21–3.06]		1.80 [1.08–2.99]	0.0234
lipid-lowering agent				
Yes	1	0.0375	-	
No	0.46 [0.22–0.96]			
Age				
< 75 years	0.44 [0.29–0.67]	0.0001	-	
≥ 75 years	1			
Grade of differentiation				
Grade 1 and 2	0.31 [0.18–0.51]	<0.0001	-	
Grade 3	1			
Presence of treatments			-	
Yes	1	0.0004		
No	3.31 [1.70–6.43]			

HR: hazard ratio; 95% CI: 95% confidence interval; p for Wald test of the null association.

## Discussion

In Martinique, between 2012 and 2016, 163 new cases of endometrial cancer were identified, reaching an average of 33 new cases per year. In our study, type II endometrial carcinoma represented 40% of our population, while UPSC, UCS and UCCC represented respectively 25%, 12%, 3% of endometrial cancers (ECs). In comparison with the literature, in which the proportion of type II cancers is usually reported around 20%, while UPSC, UCS and UCC represented respectively 10%, 5%, 3% [5, 12, 20]. Our result suggests an over-incidence of these cancers in Martinique.

The three-years DFS seems to correlate with the literature. Unfortunately, the majority of studies evaluate it at five years, whereas in our study, we had to stop after three years because of the quality of the data and the many lost views. In contrast, five-years OS in our study is very poor for all type, types I and II. When we look at the literature, it is around 71% to 76% for all types and stages combined [1, 4, 21]. For type I and II, we find five-years OS at 85.6%, and at 58.8%. However, these results on overall survival should be taken with caution because when we look at Table 4 (Extended OS curve at five years), we have a lot of censored data from a follow-up at 3.6 years: With OS at 3.6 years there is 8 censured patients or 5%. With an OS at 5 there is 40 censured patients or 25%. This is a real limit of the follow-up at five years.

**Table 4:** Overall survival in endometrial cancer patients in Martinique.

Characteristics				
	1 year	3 years	5 years	Log-rang
	% [95% CI]	% [95% CI]	% [95% CI]	
Global	75.5 [68.1–81.4]	54.6 [46.6–61.7]	47.0 [38.9–54.7]	
Histological type				
Type 1	78.4 [68.8–85.3]	63.9 [53.5–72.6]	58.8 [47.3–68.5]	<0.0001
Type 2	69.7 [57.1–79.3]	39.4 [27.7–50.9]	25.7 [25.6–37.7]	
Stage of diseases				
Figo early stage (Figo = I)	91.0 [82.1–95.6]	76.9 [65.9–84.8]	71.0 [58.6–80.3]	<0.0001
Figo advanced stage (Figo>I)	60.8 [48.8–70.9]	33.8 [23.3–44.5]	22.0 [12.7–33.0]	
Surgery				
Yes	83.7 [75.9–89.2]	69.1 [60.1–76.4]	59.1 [49.4–67.6]	<0.0001
No	46.9 [29.1–62.8]	9.4 [2.4–22.2]	9.4 [2.4–22.2]	
Chemotherapy				
Yes	68.4 [54.7–78.8]	35.1 [23.1–47.4]	22.8 [11.5–36.4]	<0.0001
No	79.4 [69.9–86.2]	67.0 [56.7–75.4]	60.2 [49.2–69.5]	
Brachytherapy				
Yes	89.4 [79.0–94.8]	68.1 [55.5–78.0]	54.6 [39.5–67.5]	0.0051
No	65.9 [55.0–74.8]	46.6 [35.9–56.6]	40.6 [30.0–51.0]	
lipid-lowering agent				
Yes	60.0 [25.3–82.7]	20.0 [3.1–47.4]	20.0 [3.1–47.4]	0.0330
No	77.1 [69.3–83.2]	56.4 [47.8–64.2]	49.0 [40.1–57.3]	
Age				
< 75 years	82.6 [74.0–88.5]	63.3 [53.2–71.6]	55.1 [44.8–64.3]	<0.0001
≥ 75 years	59.3 [45.0–71.0]	35.2 [22.8–47.8]	26.5 [14.6–40.0]	
Grade of differentiation				
Grade 1 and 2	85.5 [74.0–92.2]	74.2 [61.4–83.3]	66.7 [51.9–77.9]	<0.0001
Grade 3	67.0 [56.4–75.7]	40.7 [30.6–50.5]	30.3 [20.4–40.7]	
Presence of treatments				
Yes	75.2 [65.8–82.4]	50.5 [40.6–59.6]	41.4 [31.1–51.4]	0.2005
No	77.8 [62.6–87.4]	61.6 [45.6–74.2]	54.9 [38.2–68.8]	

When we look at each histological subtype of type II, several studies report five-years survival rates ranging from 30% to 50% in stage I patients treated for UPSC, compared to 80–90% in stage I patients treated for EEC [5, 22, 23]. Hamilton et al. report 40% in all stage of UPSC [6]. Matthews and al. report five-years OS rates ranging from 18% to 27% for patients with UPSC, but likely due to the clinical observation that approximately 60–70% of women with UPSC present with disease outside the uterus [17]. For all stages of carcinosarcoma, the prognosis for these tumors is poor, with a median disease-free survival (DFS) of approximately 12 to 16.4 months and a median overall survival (OS) of 21 to 72.0 months [20, 24], as well as a five years disease-free survival rate of 40.4% and overall survival (OS) rate of 53.6%, [25]. The five-years survival rate is estimated at 63% for UCCC [14].

This poor prognosis could be explained by the fact that our ECs are diagnosed at too advanced stage of the disease. Indeed, 49% of all types and 70% of type II are diagnosed at a FIGO stage ≥ II. In Lachance et al. study, advanced stage diagnosis accounts 34% for all ECs [26]. According to the FIGO annual report, 46% of UPSC diagnosed are at stages II to IV, compared to only 21% of EECs [11]. This histologic type, often diagnosed at advanced stages, has a high risk of recurrence, metastasis and death, even when diagnosed at early stages. In contrast with the early stages of endometrioid endometrial carcinomas (EEC), UPSC are described as having a poor prognosis even at early stages [5]. Then, Harano et al. found that 53% of carcinosarcomas are diagnosed at a FIGO stage ≥II [20].

The age of the population at diagnosis would also explain the poor survival, since 67% of ECs are diagnosed at over 65 years of age and 33% at over 75 years of age. The median age in our study population was 69 years (67 years for type I and 72 years pour type II). A series of 396 patients with endometrial cancer, showed that the older patients are more likely to have aggressive papillary serous histology, higher grade tumors, and advanced stage disease [26]. In our study, we found that 31% of the ECs in patients over 75 years of age were UPSC, which is consistent with other studies in which UPSC was reported to account for 22% of ECs in patients > 75 years of age and 3% in patients < 45 years of age [26]. As such, UPSC would be a pathology of the elderly subject. UCS increase in incidence from age 50 onwards reaching a maximum at age 75. The mean age at diagnosis is 62 to 67 years [10, 20].

Finally, the histopronostic grade of our EEC could explain the poor survival. In our study 25% of the EEC are grade 3 and several studies found that grade 3 endometrioid cancers, UPSC and UCCC share similar clinical and immuno-histochemical features, as well as poor survival [22, 27].

Martinique is a French ultra-marine department with a health system similar to that of the mainland in terms of means and access. However, our insularity imposes certain constraints: lack of certain specialists, especially gynecologists, with many patients who are not monitored. There is a long delay between the diagnosis of endometrial cancer and the first treatment due to an overloaded hospital circuit. Patients are treated according to international guidelines. The only limitation is the access to brachytherapy which does not exist on the island but patients are easily transferred by plane to mainland France for the procedure. However, some patients refuse the treatment because of the geographical distance. Finally, from a socio-cultural point of view, traditional plant-based medicine is very much a part of the Martinique population’s customs, often leading to long delays in diagnosis and therefore to diseases being diagnosed at advanced stages. Concerning the over-representation of type II, we have no demographic explanation. There is no reason why type I should be under-diagnosed compared to type II, as the sampling methods are the same and centralized.

Overall, we report a higher prevalence of type II cancers in our population, particularly UPSC and UCS, with poor prognosis. However, it is necessary to note that the incidence studies found in the literature may be outdated, as there has been an overall increase in the incidence of endometrial cancers since these data were published. The major bias of our study, in addition to be a retrospective study, is that the size of our population does not allow us to study each histological subgroup of type II. Moreover, type II EC are more common in older subjects, and the population in Martinique is ageing. Nevertheless, ethnic studies report that the disparity in survival rates in relation to ethnicity in endometrial cancers may be partly due to the high incidence of UPSC in African-American populations. According to an early study report, papillary serous and clear cell cancers were significantly more common in blacks, with 88% of papillary serous and 77% of clear cell cancers occurring in blacks [17].

Similarly, one study reported lower survival in patients of African descent compared with patients of Caucasian descent despite comparable surgical and systemic treatments and after matching histologic types, suggesting that other factors also contribute to this survival disparity [18]. Another more recent American study found an increase in all-cause mortality and cancer-specific mortality in women of African descent compared with Caucasian patients, after adjusting for socio-demographic characteristics, disease presentation, and surgical treatment [28].

Endometrial carcinomas (ECs) are heterogeneous from a genetic standpoint. Over the last decade it has become increasingly apparent that ECs are a heterogeneous group of tumors not only in terms of histology, biology, and clinical behavior, but also with respect to their genomic landscape [29]. The classification of endometrial carcinoma has evolved over time, with the aim of more precisely predicting patient prognosis and guiding management, making the dualistic view proposed by Bokman too simplistic in light of recent advances. For example, Grade 3 endometrioid cancers share characteristics of type I and type II cancer groups and have not been classified in either. This distinction has implications for surgery, choice of adjuvant therapy, follow-up and patient counselling. In addition, a clear recognition of poor prognosis associated with type II cancers may also lead to referral to specialized gynecological cancer centers. Several studies found that grade 3 endometrioid cancers, UPSC and UCCC share similar clinical and immuno-histochemical features, as well as poor survival. As such, grade 3 ECs are best characterized as type II cancers [22, 27, 30]. Access to the genetic characteristics of tumors may therefore enable the optimization of patient management to improve therapeutic responses.

Today, Next-Generation-Sequencing (NGS) technologies allow for the analysis whole human genome at such a speed that it opens new study prospects [31]. Knowledge of the genetic characteristics of tumors may enable the optimization of patient management in order to improve therapeutic responses. The Cancer Genome Atlas (TCGA)-based classification of endometrial carcinoma has shown promise in refining endometrial carcinoma classification and more accurately reflecting patient outcome. Endometrial carcinoma is classified into four categories: POLE ultra-mutated, microsatellite instability (MSI) hyper-mutated, copy number low and copy number high [32, 33].

The Martinique population being mixed, it presents different specificities and a particular genetic heritage. Moreover, the endogenous and exogenous factors are different, making comparisons with a population of Caucasian or African descent impossible. Therefore, there is a real interest in studying this population. For example, a study conducted in 2016 by Leduc and al. exploring the incidence rates of *EGFR* mutations in patients with non-small cell lung cancer of Caribbean origin found a higher rate of a mutation predictive of response to a particular targeted therapy [34]. In view of the high incidence and mortality rates of endometrial cancer in people living in Martinique, as well as the genotypic specificities in this population, it seems important to characterize the molecular profile of endometrial cancer in Martinique. To this end, a pilot study named “ENDO-NGS” is currently underway in Martinique, which aims to identify actionable mutations through the mapping by next-generation-sequencing (NGS) mapping of type II endometrial carcinoma in patients residing in Martinique (December 04, 2018 under the n° NCT03766672). This mapping should allow to assess of the proportion of type II endometrial cancers with a molecular profile comparable to those described in the literature. It could enable the identification of a subtype with a different molecular profile that would characterize the people living in Martinique and explain the excess mortality of these cancers. Today, the access to this information seems fundamental to rapidly enable the optimal management of patients in Martinique. The identification of the genetic alterations responsible for the development of type II endometrial cancer will make it possible to define the best therapeutic approach and to identify the patients who may participate in clinical trials for targeted therapy.
